# Supplementary material for: Disintegration of the NuRD Complex in Primary Human Muscle Stem Cells in Critical Illness Myopathy
Source: Int J Mol Sci. 2023 Feb 1;24(3):2772. doi: 10.3390/ijms24032772 (PMC9916927; doi:10.3390/ijms24032772)
Supplement: Supplementary file 1 [file ijms-24-02772-s001.zip › ijms-2142769-supplementary.pdf]

SUPPLEMENT

Title:

Disintegration of the NuRD complex in primary human muscle stem cells in critical illness myopathy.

Figure S1

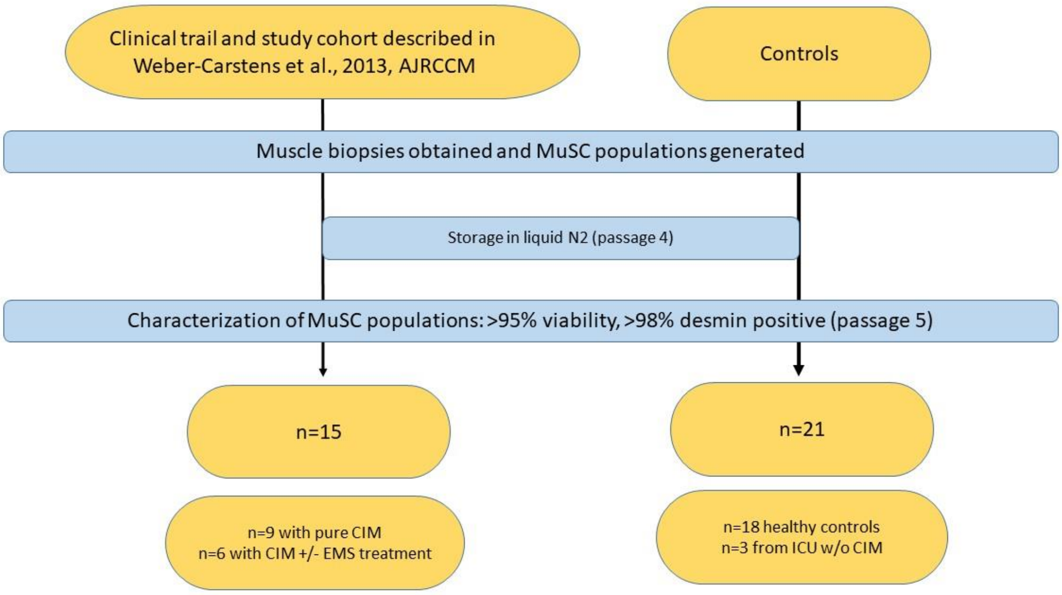

Figure S1. Study design. Muscle biopsies were obtained from n=30 ICU-patients (with and without CIM) in study cohort described in detail by Weber-Carstens et. al, 2013. Only some of the patients or legal proxies had agreed to the isolation of MuSC (12/30 patients). Of these 12 patients, n=3 underwent EMS, of which n=6 cell populations were obtained (n=3 cell populations from stimulated muscle and n=3 cell populations from nonstimulated muscle). None of the biopsied individuals had previous evidence of neuromuscular disorder and no history of critical illness. CIM-MuSC and control MuSC were isolated in the same period of time.

Figure S2

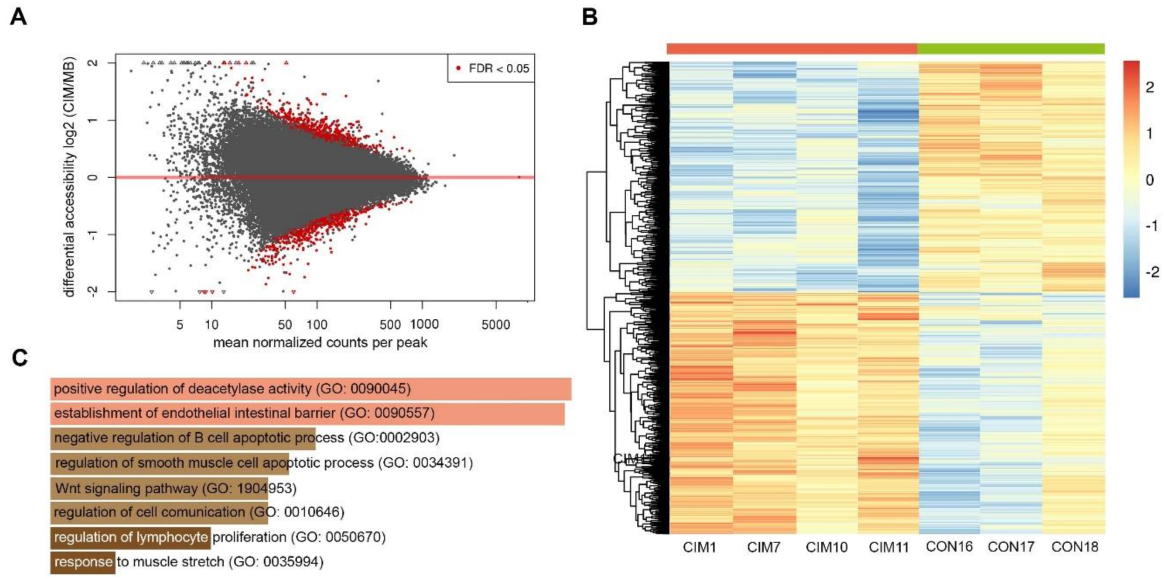

Figure S2. **ATAC-Seq analysis**.

(**A**) The MA plot shows the log2 fold changes versus the mean of normalized counts allows to inspect the results of the differential analysis. (**B**) Heatmap of row normalized counts of the 1035 differential peaks between CIM and control MuSC. (**C**) Enrichr Biological Process of more closed peaks (inactive genes) in CIM-MuSC versus control. The genes involved in positive regulation of deacetylase activity are affected.

Figure S3

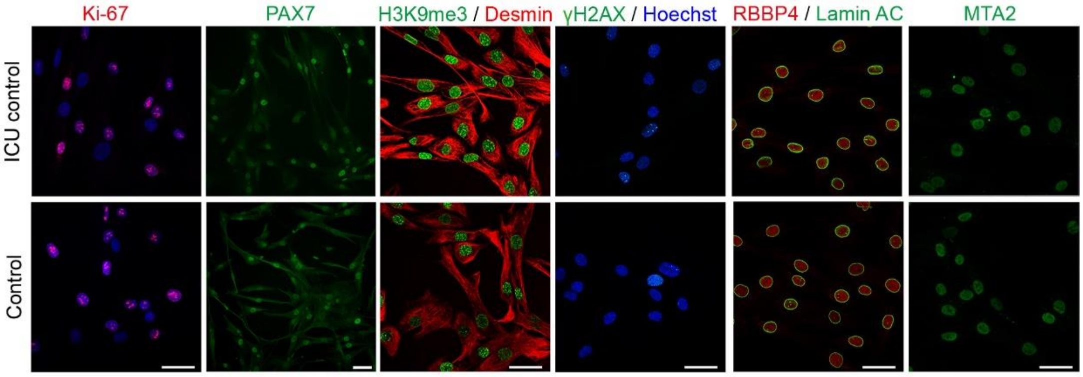

	% of positive myonuclei (mean ± SD)						
	Ki-67	PAX7	H3K9me3	γH2AX>3 foci	RBBP4	MTA2	n
ICU control	80 ± 11	39 ± 14	80 ± 11	3 ± 2	92 ± 8	95 ± 6	3
Control	80 ± 4	50 ± 18	81 ± 7	2 ± 1	96 ± 3	94 ± 3	8

Figure S3. Non-CIM-MuSC isolated from ICU patients behave like healthy controls.

Immunofluorescence staining for Ki-67, PAX7, H3K9me9, γH2AX, MTA2, RBBP4. Scale bar 50 µm. Data are presented as mean ± SD.

Figure S4

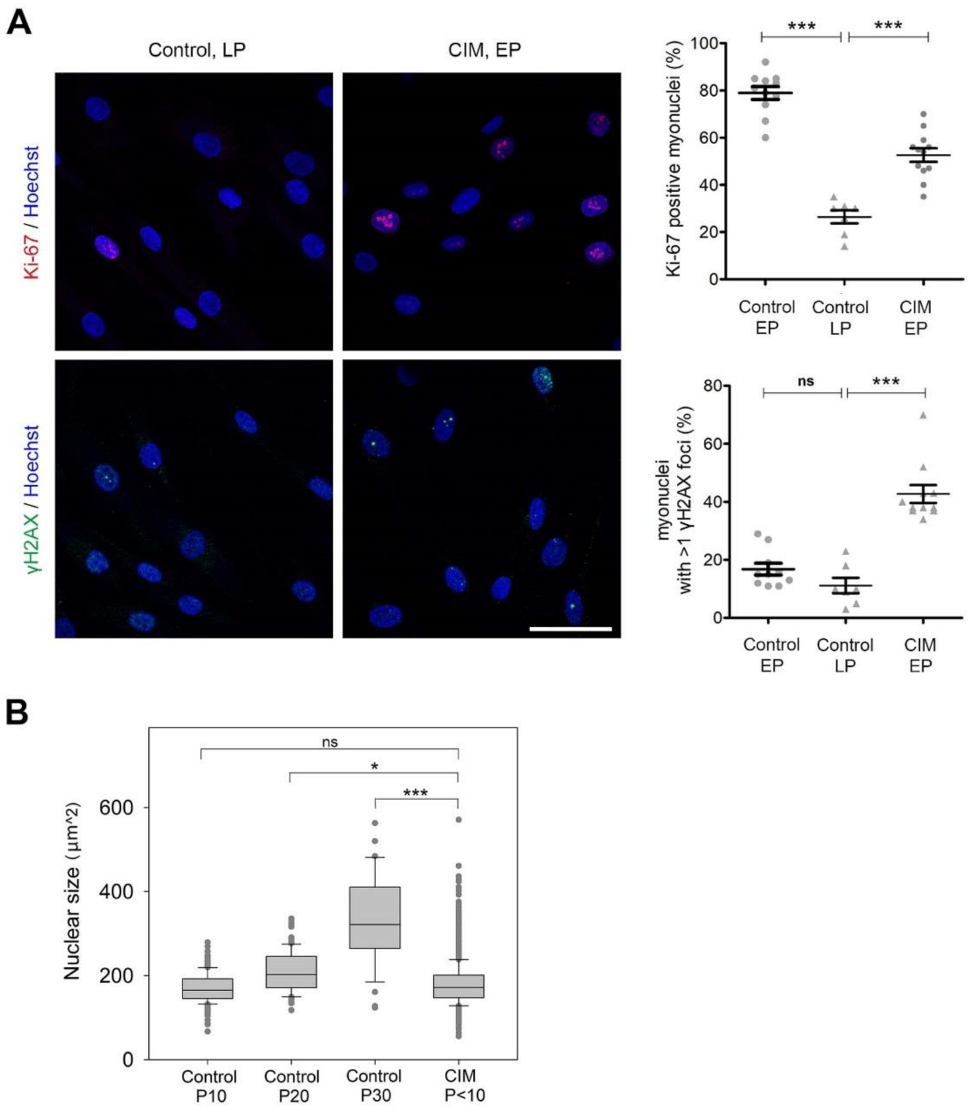

Figure S4. Comparison of CIM-MuSC with healthy, aged MuSC.

(**A**) Proliferation rate and dsDSBs in early passage (P<10, EP) (n=11 different cell populations) and late passage (P>15, LP) (n=7 different cell populations) in normal control MuSC, and in CIM-MuSC (n=12 different cell populations). Immunofluorescent staining for Ki-67 (*red*), γH2AX (*green*), Hoechst (*blue*). (**B**) Quantification of nuclear size of control healthy and CIM-MuSC at different culture passages (P). Three different control MuSC populations were analyzed at P=10, 20, 30 and four different CIM-MuSc populations at P<10.

Data information: In (A), scale bar for all images: 50µm. Data are presented as mean ± SEM. At least 200 nuclei were analyzed/cell population. In (B), nuclear size was evaluated by Image J 'Analyze Particles' function with images of DAPI stained cells. At least 100 myonuclei/cell population were analyzed, except control P30 had less cells available (n=37). Plots were generated by sigmaplot. In (A,B), Mann-Whitney-U test were used. ***p<0.001.

Table S1. Identification of cell population usage for study.

Patient	Sex, age	MuSC using for:							
		IF	WB	RNA-Seq	Atac-Seq	qPCR	dsDNA breaks analysis	nuclear size	transplant
CIM 1	m/67	+	+	+	+	+	+		+
CIM 2	m/74	+	+	+		+	+		
CIM 3	m/64	+	+	+		+	+		
CIM 4	m/53	+	+	+		+	+	+	
CIM 5	f/48	+	+			+			
CIM 6	m/67	+							
CIM 7	m/41	+			+				
CIM 8	m/36	+							
CIM 9	m/54	+						+	
CIM 10	m/41	+			+				
CIM 11	m/42	+			+				
CIM 12	m/67	+						+	
ICU CON 1	m/18	+							
ICU CON 2	m/50	+							
ICU CON 3	f/ 69	+							
CON 1	m/33	+	+			+			
CON 2	m/18	+							
CON 3	m/50	+	+	+		+			
CON 4	m/48	+	+	+		+			
CON 5	m/66	+	+						
CON 6	m/47	+		+		+			
CON 7	f/48	+	+			+			
CON 8	m/57	+	+						
CON 9	m/44	+							
CON 10	f/51	+							+
CON 11	f/48	+					+		
CON 12	m/34						+		
CON 13	m/58						+		
CON 14	m/20		+				+		
CON 15	m/68						+		
CON 16	m/37				+				
CON 17	f/28				+				+
CON 18	m/35				+				

Table S2. Clinical characterization of control-MuSC donors.

Cell population	Donor gender/age	Diagnosis	CK	Muscle histology
CON1	m/33	Myalgias, myositis excluded	2x elevated	normal
CON2	m/18	Myalgias	normal	normal
CON3	m/50	Myalgias	normal	normal
CON4	m/48	Myalgias	normal	normal
CON5	m/66	Myalgias	normal	normal
CON6	m/47	Myalgias	normal	normal
CON7	f/48	Myalgias	normal	normal
CON8	m/57	Myalgias	normal	normal
CON9	m/44	Myalgias	normal	normal
CON10	f/51	Myalgias, myositis excluded	1.5x elevated	normal
CON11	f/48	Myalgias	normal	normal
CON12	m/34	Myalgias	normal	normal
CON13	m/58	Myalgias	normal	normal
CON14	m/20	Myalgias	3x elevated	normal
CON15	m/68	Statin-induced myalgias	normal	normal
CON16	m/37	Myalgias	normal	normal
CON17	f/28	Myalgias	normal	normal
CON18	m/35	Carrier	normal	normal

Table S3. Primary antibodies.

Antibody	Company	Catalog #	Dilution IF[1]	Dilution WB[2]	Blocking solution WB
alpha-Tubulin	Sigma-Aldrich	T5168		1:1000	4% milk in TBS
beta-Tubulin	Abcam	ab6046		1:1000	4% milk in TBS
Desmin	DAKO	M0760	1:100		
Desmin	Abcam	ab8592	1:100		
HDAC1	Santa Cruz	sc-7872	1:200		
Histone 1	Abcam	ab125027	1:100	1:500	4% BSA in TBS
Histone 3 (trimethyl K9)	Abcam	ab8898	1:10000	1:2000	4% BSA in TBS
Ki-67	Thermo Fisher	MA514520	1:300		
Lamin AC	Abcam	ab108595	1:2000		
MTA2	Santa Cruz	sc-9447	1:200	1:500	4% milk in TBS
Myosin Heavy Chain (fast)	NovoCastra	NCL-MHCf	1:300		
Myosin Heavy Chain (slow)	NovoCastra	NCL-MHCs	1:20		
PAX7	Santa Cruz	sc-81648	1:200		
phospho-H2A.X	Millipore	05-636	1:200	1:1000	4% BSA in TBS
RBPP4 (RbAp48)	Abcam	ab488	1:400	1:500	4% milk in TBS
Spectrin	Leica	NCL-SPEC1	1:100		

[1]IF = immunofluorescence; [2]WB = Western blot

Table S4. Secondary antibodies.

Antibody	Company	Catalog number	Dilution	Application
Alexa 488	Invitrogen	A11055	1:500	[1]IF
Alexa 488	Invitrogen	A11001	1:500	IF
Alexa 488	Invitrogen	A11008	1:500	IF
Alexa 568	Invitrogen	A11031	1:500	IF
Alexa 568	Invitrogen	A10042	1:500	IF
Alexa 568	life Technologies	A11057	1:500	IF
Alexa 568	life Technologies	A11036	1:500	IF
Alexa 647	Invitrogen	A21469	1:500	IF
Alexa 647	Invitrogen	A21245	1:500	IF
Alexa 647	Invitrogen	A21236	1:500	IF
Alexa 647	Invitrogen	A31571	1:500	IF
800	Licor	92632214	1:5000	[2]WB
800	Rockland	611-745-127	1:5000	WB
800	Rockland	610-732-124	1:5000	WB
700	Rockland	611-730-127	1:5000	WB
700	biomol	21056	1:5000	WB
HRP	GE Healthcare	NA934	1:2000	WB
HRP	Invitrogen	626520	1:2000	WB

[1]IF, immunofluorescence; [2]WB, Western blot

Table S5. Primer used for qPCR analysis.

Gene	Forward primer	Reverse primer
HIST1H3D	GCCAAGGCAGGGTTTAGAAG	TGCTTGCGTGGCGCTTT
HIST1H2AE	GCAACGACGAGGAGCTAAA	TCCGTCTTCTTAGGCAGCAA
HIST1H3C	AGGACTTCAAAACCGACCTG	TTAGCGTGAATAGCGCACAG
PAX7	GATTCCCTTTGGAAGTGTCC	ACTATCTTGTGGCGGATGTG
CyclophilinA	CGCCGAGGAAAACCGTGTACTATT	GACCTTGTCTGCAAACAGCTCAAAG
GAPDH	GAAGGTGAAGGTCGGAGTC	GAAGATGGTGATGGGATTTC
RPL13a	CGTGCGTCTGAAGCCTACA	GGAGTCCGTGGGTCTTGAG

Table S6. ATAC-Seq. Mapping Stats.

Sample	Total Number of Reads	Total Number of paired Reads	Number of Reads not aligned	Number of Reads aligned once	Number of Reads aligned multiple times	Overall alignment rate (%)	Number of Reads after Filtering	Non-Redundant Fraction (NRF)
CIM 1	105’967’776	105’967’776	2’827’551	69’804’678	33’335’547	97.33	67’592’069	0.638
CIM 7	101’145’206	101’145’206	2’880’501	64’893’460	33’371’245	97.15	33’045’906	0.327
CIM 10	117’499’450	117’499’450	2’762’445	75’236’211	39’500’794	97.65	84’786’234	0.722
CIM 11	122’654’119	122’654’119	3’320’867	80’945’354	38’387’898	97.29	60’346’667	0.492
Control 16	128’259’988	128’259’988	4’420’662	82’816’488	41’022’838	96.55	47’673’488	0.372
Control 17	113’114’747	113’114’747	3’547’167	73’857’109	35’710’471	96.86	60’233’550	0.532
Control 18	123’349’087	123’349’087	4’219’323	80’326’238	38’803’526	96.58	53’263’782	0.432

Table S7. ATAC-Seq. Peak Calling Stats.

Sample	Number of Peaks	Fraction of Peaks in Blacklist (%)	Fraction of Reads in Peaks (%)	Fraction of Reads in Blacklist (%)
CIM 1	42’350	2.413	4.063	2.064
CIM 7	37’533	2.363	5.925	3.144
CIM 10	162’417	0.687	14.159	1.402
CIM 11	63’012	1.652	7.640	2.230
Control 16	72’156	1.457	10.811	2.821
Control 17	78’029	1.466	9.927	2.670
Control 18	41’950	2.410	5.448	2.644
CIM group	340’433	0.570	-	-
Control group	227’232	0.743	-	-

Table S8. ATAC-Seq. Consensus Stats.

Group	Number of Peaks in Group Consensus	Fraction of Peaks in Consensus Set (%)	Number of unique Peaks for Group
CIM	34’520	10.140	1’275
Control	53’869	23.707	6’919

SUPPLEMENTAL METHODS:

ATAC-Seq

Generate count matrix over consensus peaks: The number of reads overlapping the derived consensus peaks for each sample has been acquired using the bedtools module multicov, where only reads with a minimum mapping quality score of 10 have been counted (version 2.27.1, '-q 10').

Post processing removal of the unwanted variations: Upon initial principal component analysis (PCA) using the raw count data, we noticed that patient age was the most discriminative feature (PC1: 87.3%), whereas the health condition of the patients seemed to be the second most distinctive information. This indicated the need for normalization techniques which account for the in-group variation and estimate the influence of covariates, e.g. the age of the respective patient, which impact in the chromatin dynamics of patients irrespective of the health condition. Due to similar characteristics of the sequencing data, like genome wide distribution and variable length enrichment, we decided to use methods designed for RNA-Seq, especially for differential expression (DE) analysis. We used an approach called RUVSeq which identifies known or unknown factors of "unwanted variation", e.g., batch, library preparation, and other nuisance effects, using the between-sample normalization methods proposed in [45]. The influence of the identified factors is estimated per patient and can be accounted for in the differential analysis framework. In a recent study Koberstein et. al. analyzed how learning alters chromatin accessibility in the mouse hippocampus and showed that this method can be successfully applied to epigenetics data to correct for unwanted variability [46].

Differential analysis: The subsequent differential analysis was performed on the raw read counts of the 74292 consensus peaks with DESeq2 (version 1.20.0) using three factors of unwanted variation derived from RUVr as covaritiates in the design formula. We performed Wald test comparing health conditions CIM vs Control followed by multiple testing correction of the p-values using Benjamini-Hochberg method, then we employed a log2 fold change threshold of 0.1

to account for background signal and a significance cutoff of 0.05 for the adjusted p-values to identify 1128 differential sites (more open: 660, 0.89%; more closed: 468, 0.63%).

The following post processing steps have been performed using in-house scripts written in R heavily employing software provided through the Bioconductor infrastructure, especially using the software for computing and annotating genomic ranges [47] and graphics have been created using the ggplot2 R package (version 3.0.0) unless otherwise stated.

Generation of fragment size distribution: The fragment lengths were acquired by extracting the value of the TLEN field of the alignments from all proper read pairs that were marked as primary alignments and not being duplicate. Then the fragment length was plotted against the read density to see the expected open chromatin region (peak at < 100bp) followed by nucleosome phasing pattern (peak every ~ 200 bp).

Plot fragment size classes at TSS: The fragment lengths were extracted from reads mapping within 4kb (+/- 2kb) of transcription start sites of Ensembl annotated genes (Release GRCh37.87). TSS specific fractions of template lengths were acquired for all samples and plotted averaged over all samples per group (Figure 2C in the middle), where error bars show the standard deviation of fractions per group. The bar graph shows the fraction of template length occurrences relative to all extracted template lengths.

Plot fragment sizes intergenic classes: The fragment lengths were extracted from reads mapping to non-gene covered regions of the genome (intergenic regions). Intergenic fractions of template lengths were acquired for all samples and plotted averaged over all samples per group (Figure 2C on the right), where error bars show the standard deviation of fractions per group. The bar graph shows the fraction of template length occurrences relative to all extracted template lengths.

Removal of unwanted variations: In detail, the RUVr approach were followed as described in the package manual, first we performed upper-quartile (UQ) normalization of the data and then we

estimated residuals from an initial generalized linear model (GLM) regression of the counts on the covariates of interest to estimate the factors of unwanted variation. The factors of unwanted variation were validated by comparing them against known covariates as for example age of the patient donor.

Differential analysis with DESeq2: The differential analysis was performed with DESeq2 (version 1.20.0) using the design formula, based on the three factors of unwanted variation derived from RUVr. The MA plot showing the log2 fold changes versus the mean of normalized counts allows to inspect the results of the differential analysis (Figure S2A). The row normalized counts of differential peaks indicate equal group sizes for more open and more closed peaks. The heatmap have been created with the R package pheatmap (version 1.0.10) (Figure S2B).

Effect on housekeeping genes: Furthermore, we were interested in the effect that this normalization has on the counts of stable genes, like housekeeping genes. We downloaded a list of housekeeping genes derived from the analysis of next-generation sequencing (RNA-Seq) data (https://www.tau.ac.il/~elieis/HKG/) and extracted their promoter regions (+2kb,-200b from TSS) from the UCSC knownGene annotation for hg19. Next, the genes with overlapping peaks were filtered out and raw counts of mapped peaks against the RUVseq normalized counts were compared. We observed a constant smoothing of the count values upon removal of more factors of unwanted variation.

Genomic Feature Annotation: A basic annotation of the peak sets were performed using the annotateWithGeneParts function of the genomation package to assign peaks to genomic features and found that most of the peaks lie in non-coding regions like introns or intergenic regions.

Enrichment Analysis**:** Since many of our peaks of interest are located in non-coding areas of the genome distal to promoters of genes, we use rGREAT (version 1.12.1) a client to the Genomic Regions Enrichment of Annotations Tool (GREAT) (version 3.0.0) to analyze the peaks as long range acting cis-regulatory elements. Annotation was performed Regulatory domains of genes are

defined based on curated regulatory domains and GREAT's Basal plus extension model, where each gene is assigned a minimal regulatory domain spanning 5 kb upstream and 1 kb downstream of the TSS regardless of neighboring genes, which is then extended in both directions to the minimal regulatory domain of the nearest gene or up to 1000 kb. GREAT assigns the peaks to these regulatory domains and performs a genomic region–based enrichment test against the whole Genome allowing to assign a functional significance to the identified long-range gene regulatory domains. The results of the enrichment analysis were filtered for having an adjusted binomial p-value (BinomQ) below 0.1 and an adjusted hypergeometric p-value (HyperQ) below 0.5 to account for biases in gene regulatory domain size and correct for the number of genes located in regulatory domains. These filters removed any results for the enrichment analysis of the more open peaks but highlighted significant gene ontology terms for the more closed regions.

Enrichr: To validate our findings, we used the webservice Enrichr at http://amp.pharm.mssm.edu/Enrichr to analyze our differential peak sets. Enrichr queries a large collection of diverse gene set libraries given BED files of the regions of interest. The sets of more closed and more open peaks were queried separately and the ontology analysis was consistent with GREAT's results, apart from a change in ranking of the terms due to a different scoring method (Figure S2C).
